# A novel outreach approach for identification of familial hypercholesterolemia: Interview-based formative evaluation to improve healthcare access and quality

**DOI:** 10.1016/j.pecinn.2025.100407

**Published:** 2025-06-02

**Authors:** Rachel C. Forcino, Terry Sturke, Mary P. McGowan, Amanda N. Perry, Shoshana H. Bardach, Vikrant S. Vaze, Kerrilynn C. Hennessey

**Affiliations:** a Department of Population Health, University of Kansas School of Medicine, Kansas City, KS, USA; b Family Heart Foundation, Fernandina Beach, FL, USA; c Heart and Vascular Center, Section of Cardiovascular Medicine, Dartmouth Hitchcock Medical Center, Lebanon, NH, USA; d The Dartmouth Institute for Health Policy and Clinical Practice, Dartmouth Geisel School of Medicine, Hanover, NH, USA; e Thayer School of Engineering at Dartmouth, Hanover, NH, USA

**Keywords:** Familial hypercholesterolemia, Formative evaluation, Direct-to-patient outreach

## Abstract

**Background:**

Familial hypercholesterolemia (FH) is a genetic condition which elevates cholesterol levels and increases risk of premature cardiac events. Medical treatment greatly reduces those risks. However, in the United States, FH is markedly underdiagnosed. We aimed to design and evaluate direct outreach and referral to specialty care for patients with an elevated risk of FH identified through application of a machine learning model and expert review of the electronic health record in a rural United States health system.

**Methods:**

Two sets of interviews: (1) seeking advice for designing outreach from a convenience sample comprising health professionals and members of the public both with and without FH and (2)) evaluating the outreach with a convenience sample of health professionals and patients who received the outreach. Two researchers conducted each interview. Thematic analysis included investigator triangulation.

**Results:**

We conducted 15 pre-outreach interviews and 32 post-outreach interviews. Most members of the public felt the outreach should be initiated by the patient's primary care clinician, while health professionals recommended outreach directly from a lipid specialist after notifying the primary care clinician. Outreach ultimately included primary care clinician notification; a mailed letter from lipid specialists to the patient indicating partnership with primary care; a message sent through the online patient portal; and a telephone call(s) from a lipid specialist to the patient. Phone calls were most impactful in prompting a clinical evaluation for FH. We identified 4 themes: (1) Both patients and clinical team members supported direct-to-patient outreach about FH; (2) Phone calls from lipid specialists to patients were considered high-value; (3) The importance of primary care team member involvement was perceived differently between pre- and post-outreach phases; and (4)) Outreach had a broader impact beyond the individual patients reached, including family screening.

**Innovation:**

This study provides key insights into the acceptable design and use of machine learning and electronic health record data for direct-to-patient outreach.

**Conclusions:**

Partnership with the target population led to direct-to-patient FH outreach that was acceptable to most recipients. High-touch engagement by lipid specialists included repeat phone calls, which maximized patients' response but are unlikely to be sustained in routine practice.

## Highlights

• Targeted phone call outreach about clinical evaluation for familial hypercholesterolemia (FH) was acceptable to patients.
• Repeat phone calls by lipid specialists maximized uptake of FH clinical evaluation in our project.
• We identified challenges related to sustaining this repeat phone call approach in routine practice.

## 1 Introduction

Familial hypercholesterolemia (FH) is a genetic condition which elevates cholesterol levels and increases risk of early cardiac events [1]. Among people with FH, initiating cholesterol-lowering treatment early in life and before the onset of cardiovascular disease improves health outcomes [2,3].

However, in the United States, FH is diagnosed in only 20 % of the affected population of about 1.3 million people [4,5]. FH often remains undiagnosed for a variety of reasons, including a lack of training about the condition among both primary care and cardiology clinicians and a lack of systems to identify the people most at risk [6].

To improve rates of FH diagnosis, the Family Heart Foundation (FHF) created a machine learning algorithm called FIND-FH® [7]. The algorithm draws on dozens of deidentified data elements from electronic health records to predict which patients are most likely to have FH. A previous effort to implement FIND-FH within a health system failed to substantially improve the rate of FH evaluation and diagnosis due to several key barriers – most notably a lack of patient and primary care involvement in program design and patient outreach [8].

In this quality improvement project, we aimed to address these previously identified barriers by designing and evaluating direct outreach and referral to specialty care for patients who were flagged as having an elevated risk of FH through application of the FIND-FH machine learning model [7] and expert review of patients' electronic health records at Dartmouth Health, a rural health system in the United States.

## 2 Methods

### 2.1 Design

We conducted a qualitative, semi-structured interview study designing and evaluating direct-to-patient outreach about FH. We used a participatory approach, interviewing a diverse range of people who may be interested in and/or potentially impacted by our outreach and incorporating their perspectives into the processes and materials used in this project. We report the project according to the Revised Standards for Quality Improvement Reporting Excellence (SQUIRE 2.0) and the Standards for Reporting Qualitative Research (SRQR) [9,10]. The Dartmouth Health Institutional Review Board designated project activities as not human subjects research.

### 2.2 Context/setting

We conducted this quality improvement project in Dartmouth Health, a rural health system in the northeast United States between January 2023 and April 2024 [11]. Patient eligibility for outreach was determined by project team physicians' review of patients flagged by the machine learning algorithm (FIND-FH [7]) in the health system's electronic health record (Epic Systems). Physician project team members evaluated flagged patients' medical records for factors that precluded outreach: an existing FH diagnosis, prior visits to the health system's lipid clinic, insufficient information (e.g., no reported LDL-C levels, no information on personal/family history of cardiovascular disease) or otherwise low likelihood of FH (e.g., no evidence of substantially elevated LDL-C levels or personal/family history of cardiovascular disease), relocation outside the health system's service area, alternative medical priorities impeding a new focus on FH, and death. Project team members contributed expertise in lipid disorder diagnosis and management, lived experience of FH, systems engineering, machine learning, healthcare business analytics, quality improvement, and health services research. In this project, outreach letters, messages, and phone calls from physicians were unreimbursed, while any subsequent visits and testing (e.g., lipid panel, lipoprotein (a)) were billed through insurance for reimbursement. While not the specific focus of this outreach, lipoprotein (a) (Lp(a)) testing was offered to patients because Lp(a) elevations are known to be more common in individuals with FH than in the general public [12].

### 2.3 Participants

#### 2.3.1 Pre-outreach design phase

To inform outreach design, we recruited convenience samples of (1) members of the public with clinically diagnosed FH, (2) members of the public without FH, (3) primary care clinicians at the participating health system, including primary care administrators/leadership, and (4) cardiology clinical and administrative team members at the participating health system. We did not exclude interview participants on the basis of age or any other demographic criterion.

#### 2.3.2 Post-outreach evaluation phase

To evaluate the outreach, we recruited convenience samples of (1) health system patients who received the outreach, (2) primary care clinicians whose patients were evaluated through the project, (3) physicians specializing in lipid management, and (4) cardiology clinical and administrative team members (e.g., schedulers) at the participating health system. We did not exclude interview participants on the basis of demographic criteria.

### 2.4 Data collection

Two interviewers conducted each interview, with one recording detailed field notes throughout. Prior to each interview, the interviewers explained the nature and goals of the quality improvement project. Interviewers periodically assessed thematic saturation through writing memos and discussion, continuing data collection until they reached consensus that thematic saturation was achieved.

### 2.5 Data analysis

A single analyst (ANP) independently coded interview field notes using a deductive framework approach, guided by the topics in the interview guide [13]. By reviewing all field notes, the single independent analyst was able to deeply immerse herself into the content, enabling a comprehensive review of feedback. To enhance rigor and reliability, two additional members of the research team met regularly with the primary analyst to systematically review and discuss all coding. Three total analysts (ANP, TS, RCF) reviewed interview field notes and coding in detail. Analysts were all women and included a research program manager with no personal experience of FH, a health services researcher with no personal experience of FH, and a healthcare administrator with FH. The three analysts met at the end of each project phase to propose, discuss, justify, and finalize themes.

## 3 Results

### 3.1 Participant characteristics

Table 1 summarizes interview participants over the project's design (*n* = 15) and evaluation (*n* = 32) phases. Design and evaluation phase participant samples were independent, except for the cardiology scheduler category.

**Table 1:** Participant summary.

	Design phase (n = 15)	Evaluation phase (n = 32)
Members of the public with FH	5	–
Members of the public without FH	4	–
Members of the public flagged as at-risk for FH	–	19
Primary care clinicians (including leadership)	4	8
Cardiology schedulers	1	2
Physicians specializing in lipid management	–	3
Specialty pharmacists	1	–

### 3.2 Designing the outreach program: core components

The development of the outreach program was informed by: the project's design phase interviews which included patients, potential patients, physicians, and hospital administrators; input from the FHF; and input from the interdisciplinary project team, including 3 physicians specializing in diagnosis and treatment of lipid disorders. This input emphasized the trusting relationships patients have with their primary care clinicians and a desire for a straightforward and efficient path to scheduling an FH evaluation. In crafting outreach materials, we drew on the extended parallel process model [14] and prioritized balancing the importance and gravity of downstream FH-related harms (i.e., acute cardiovascular disease) with preventing undue fear and/or anxiety among outreach recipients.

The outreach program ultimately included: (1) Notification from lipid specialist to patient's primary care clinician; (2) Letter from lipid specialist to patient sent by mail and/or an electronic message in myChart online patient portal; (3) Up to 3 phone calls from lipid specialist to eligible patients who had not yet scheduled (nor explicitly declined to schedule) a visit in response to prior outreach.

### 3.3 Evaluating the outreach program

We identified 4 major themes in our analysis: (1) Both patients and clinical team members supported direct-to-patient outreach about FH; (2) Phone call outreach from lipid specialists to patients was considered high-value; (3) The importance of primary care team member involvement was perceived differently across project phases (design vs. evaluation); and (4) The outreach program had an impact beyond the individual patients reached. Within theme 1, we identified 3 sub-themes: (1a) Only some patients considered privacy implications; (1b) Clarity is needed around costs, billing, and insurance coverage; and (1c) Family and/or personal history of early cardiac events had varied influences on how patients responded to FH outreach.

#### 3.3.1 Theme 1: Both clinical team members and the patients who received it supported direct-to-patient outreach about FH

##### 3.3.1.1 Primary care clinicians

Primary care clinicians were supportive of the outreach program. They felt it complemented their clinical practice and they wanted outreach to continue. One primary care clinician reminded us that “[PCPs] can't take care of everything for everyone. Having a second pair of eyes is wonderful.” Another primary care clinician expressed disappointment at the quality improvement project coming to an end: “It is a shame that the program isn't ongoing. I think it would pick up speed and more people [would] refer to the program.”

##### 3.3.1.2 Administrative staff

Cardiology clinic administrative staff who were impacted by the direct-to-patient outreach about FH were also supportive. Schedulers were comfortable with both the outreach and the process, finding it similar to existing duties. One scheduler explained, “It wasn't too different – it is like what I do now. I had to make sure the labs were sent to the place [the patient] wanted to go. It was like what I do usually.” Another scheduler mentioned that the outreach “gave patients important information and help,” and that involvement in the outreach and clinical evaluation process “was rewarding” work.

##### 3.3.1.3 Patients

Most patients welcomed outreach about FH, even when they did not follow up on it. One patient told us that “it is important to continue to do this outreach, especially for people like me who are too scared to deal with the reality” of addressing the cardiovascular risks that they have witnessed manifest in close family members. Another patient reported appreciating the outreach while feeling unable to follow up on it in a timely manner because of his competing responsibilities as the parent of young children. Other patients expressed appreciation for the outreach and a desire to continue seeing the project's lipid specialists, with one saying, “I wish [lipid specialist] would be my provider going forward.”

*Sub-theme 1a: Only some patients considered privacy implications*

In the project's design phase, the outreach team was concerned about how patients would perceive being identified through a new machine learning approach. However, few interview participants mentioned privacy considerations related to patient identification and outreach. One patient reacted to the outreach with surprise and “a small amount of fear,” wondering “how [his] name got in the study.” This patient then described his understanding of limited privacy in digital spaces, saying, “I sort of assumed at the outset that any information about me is out there.” Another patient recommended being “very conscious of [patients'] HIPAA rights,” referring to United States HIPAA legislation governing patient privacy and saying that he “could have been angry at [lipid specialist] having [his] health information.” Another simply found it “interesting that my file was reviewed and that I qualified for the program.” These responses reflect the novelty of direct-to-patient outreach by specialists in our health system, while also revealing a lack of existing norms surrounding this type of data use in a healthcare context in contrast to widespread digital data use in other sectors.

*Sub-theme 1b: Clarity is needed around costs, billing, and insurance coverage*

Patients, project team physicians, and cardiology clinic staff all identified a need for clarity about the out-of-pocket costs associated with FH clinical evaluation given that proactive identification and outreach are outside current norms in clinical medicine. Patients reported being unsure both whether the phone call outreach they received from physicians would be billed as a visit, and whether they would be charged for any subsequent formal telehealth or in-person visits or testing. A scheduler mentioned receiving questions from patients about whether insurance would be billed for their upcoming clinical evaluation visit. A project team physician reflected, “We had conversations about billing and I gave them information about possible costs. […] Some people were upset with the cost of lipoprotein (a) [testing]. I made some changes to the protocol so people would know what to expect regarding cost. […] It is hard when people say they want to see me but can't see me – that is really hard. These are not routine questions that we get and because we were outreaching it was difficult.” A patient also discussed the financial burdens of cascade screening, saying, “I have grandchildren and they don't have insurance. I feel that some of the burden for testing that is recommended for FH should come from the [QI project].”

*Sub-theme 1c: Family and/or personal history of early cardiac events had varied influences on how patients responded to FH outreach*

Given the hereditary nature of FH, many patients who received our outreach had family and/or personal experiences with early cardiac events. Most patients found that these experiences increased their receptivity to the FH outreach; however, in a few instances, the fear of a known family history resulted in apprehension to address the possibility of increased personal risk. One patient expressed improved understanding, saying, “It explains a lot. I had a brother who died of a heart attack at 46, my other brother had bypass and so did my mother.” Another described being “excited to have the call and thought maybe there is something new” available to support his cardiovascular health after his heart attack several years prior. Another patient “wasn't totally surprised” given that her “father and grandmother had high cholesterol,” and “was pleased that they [the health system] were concerned.” Another patient “thought there was a strong possibility that I had it given my history,” but considers herself “laid-back” and was therefore at ease with the FH outreach. For other patients, FH outreach served as a somber reminder of their family history of cardiac events. One patient describes putting the letter aside, “afraid to face reality,” but was ultimately responsive to repeated phone call outreach.

#### 3.3.2 Theme 2: Phone call outreach from lipid specialists to patients was considered high-value

Many patients did not act on the initial letter they received but responded when contacted by phone. As one patient explained, “I didn't think it was a hot issue and set [the letter] aside. Then [lipid specialist] reached out to me about a week after I got [the letter]. It intrigued me as it was the [same] doctor who called me [who mailed the letter].” In describing next steps after receiving the outreach, another patient said, “I put the letter with my bills and waited to be contacted […] I received a phone call on my home phone that I missed and then I got one on my cell phone that I took. I felt that they were really following up with something they started.” Another patient mentioned that he “had other priorities” and that “the letter sat in the things to do list until [he] got a phone call.” Patients recognized and appreciated the significant effort involved in physicians contacting them by phone. One patient explained, “there is something about that call that is caring. After all, we know that doctors don't call patients and if a doctor calls, it is important.”

In addition to their responsiveness to the phone call itself, patients valued the communication style and expertise of the lipid specialists who contacted them by phone. Specifically, patients appreciated the physicians' knowledge of the disease and of their personal health histories paired with a shared decision-making approach to further testing and treatment. One patient noted, “I value the high level of expertise that [lipid specialist] has. She is up to date, and she respects my feelings about meds, but she really knows what is better.” Another mentioned, “how [lipid specialist] explained the situation […] it was my choice. Nobody was making me do anything.” Another patient expressed, “I appreciated [lipid specialist] not pushing me to set up an appointment. I did not feel coerced. She seemed to genuinely have my interests at heart and was very thorough. I could tell she was an expert […] She didn't make me feel like a jerk if I said no.”

While patients were highly appreciative of and responsive to phone calls from lipid specialists, phone call outreach was a significant time commitment for these physicians. The physicians involved in the project said they felt this approach was effective but unsustainable in the long term. One project team physician compared outreach phone calls to typical new patient referrals, explaining that outreach calls were “a longer conversation with someone you don't know, and it takes a lot more to help them understand. This may be the first they have heard about it. […] It was many more hours than I expected.”

#### 3.3.3 Theme 3: The importance of primary care team member involvement was perceived differently across project phases

During the design phase, members of the public emphasized the importance of having members of their primary care team involved in outreach to patients at risk of FH but also acknowledged the role of a specialist in making the diagnosis. One participant interviewed during the design phase without personal experience of FH explained, “It is ideal if [the outreach] came from my PCP [primary care provider]. If not my PCP, then someone in that office.” However, primary care leadership described a primary care environment of limited resources and many demands on clinicians' time. One leader explained, “In my job, I am trying to retain providers. I am trying to make their lives bearable. If we ask them to do more, it can become unbearable. […] It is hard for a PCP to follow all the guidelines. To do so, you would need to work 17 hours a day.” Another primary care leader suggested “an FYI to the PCP” via a staff message in the electronic health record as the appropriate level of primary care involvement in this outreach program.

In the evaluation phase, most patients who received the outreach reported no interaction with their primary care clinicians in their decisions about whether to pursue clinical evaluation for FH and in any follow-up to the evaluation. When asked about primary care interaction over the course of his involvement with the project, one patient stated, “No, I don't talk to him often.” Another patient explained that she prioritizes the specific expertise of a physician specializing in lipid disorders over her preference of being contacted by her primary care clinician, saying, “My PCP has a general understanding and tells me that as long as my cholesterol is below 200 it's okay, but [lipid specialist] recommends that my numbers should be lower. It feels weird that I am contacted by someone on the side but, in fact, my PCP doesn't know as much as [lipid specialist].”

In these contrasting reflections from patients and members of the public, we identified differences between the preferences expressed in a hypothetical outreach scenario for primary care involvement (design phase) and the behaviors reported by patients who received outreach and actively faced a healthcare decision (evaluation phase).

#### 3.3.4 Theme 4: The outreach program had an impact beyond the individual patients reached

In addition to the impact of the outreach program on those patients who were identified in the electronic health record and received our outreach about FH, most patients we interviewed reported contacting their family members to share information on this genetic disorder and available testing. One patient told us, “I wanted to pass [FH information] on to family members. […] I reached out to my son and told him he needs a lipid profile done with a lipoprotein(a) test. I also told my sister and she got a lipid profile.”

Beyond patients encouraging cascade screening within their families, a few primary care clinicians referred patients with high cholesterol who were not flagged by the machine learning algorithm to the project for FH clinical evaluation. One explained that he “referred a few patients to the program and told them I recommended that they go” because “it closes the care gap” that patients sometimes experience in accessing specialty care.

## 4 Discussion and conclusion

### 4.1 Discussion

Patients and clinical team members found direct-to-patient outreach about possible FH acceptable when initiated by project team physicians after notifying the patient's primary care clinician, despite some questions about patient privacy and out-of-pocket costs. Patients found phone calls from project team physicians to be more motivating for scheduling an FH clinical evaluation than mailed letters or messages sent through the electronic health record. However, project team physicians felt that the time required for personalized phone call outreach would be unsustainable in an ongoing FH evaluation program as part of their routine duties. The impact of the outreach program extended beyond the specific patients receiving outreach, as patients shared FH information with family members and primary care clinicians referred additional patients for FH clinical evaluation.

Despite randomized controlled trial evidence demonstrating mailed informational materials to be an effective means of patient outreach for promoting uptake of lipid-focused clinical evaluation and therapy [15], our participants reported limited impact of receiving a mailed letter describing FH risk on their decision to pursue clinical evaluation. Instead, we found phone calls from physicians to at-risk patients to be a more impactful mode of direct-to-patient outreach.

Many have written about the promise of electronic health record data for health systems' continuous quality improvement [[16], [17], [18], [19]], but less attention has focused on the acceptability of this data use among patient populations. Our results highlight an ongoing need to balance health systems' enthusiasm for data-informed quality improvement with clear and meaningful communication to patients about how their personal health information is used at the health system level.

Use of algorithms and electronic health record data to identify patients at risk has the potential to improve both the uptake and the harm-benefit profile of FH screening and clinical evaluation efforts. In general, screening processes that maximize benefit while minimizing harm must target the right people (i.e., those at risk of disease that contributes to morbidity and mortality) at the right time in their possible disease trajectories to effectively intervene in service of better clinical outcomes [20,21]. Our use of a predictive model in combination with expert review by lipid specialists improved the screening process for identifying people with FH, better targeted patients for outreach, and thereby improved the harm-benefit profile of FH clinical evaluation in the targeted population.

One harm associated with clinical evaluation is the financial burden of clinic visits and testing. While our efforts to better target patients for outreach improved their possibility of benefit from FH clinical evaluation, patients interviewed in our project also described financial burdens, especially in the absence of adequate health insurance coverage. First advanced in the oncology literature [22], the idea of financial toxicity of health services is gaining recognition across clinical contexts, including in the cardiology field [23,24]. Targeting outreach efforts to those most likely to benefit from screening and clinical evaluation and continuing to share resources for financial support with patients pursuing clinical evaluation have the potential to minimize unnecessary financial burden.

#### 4.1.1 Strengths and limitations

We report the formative evaluation of a single health system's efforts to improve uptake of clinical evaluation for FH among those patients most at risk. Our results therefore may not generalize to other institutions, communities, or contexts. In this quality improvement project, we recruited convenience samples of interview participants. While we intentionally sought out patient participants who chose not to pursue FH clinical evaluation along with patient and clinical participants who we perceived to react unfavorably to our outreach program, sampling and selection biases may have resulted in underrepresentation of critical views in our dataset. Finally, we were unable to fully evaluate the downstream impact of our outreach (e.g., family communications and subsequent cascade screening).

### 4.2 Innovation

Targeted patient screening and direct-to-patient outreach for FH using a physician-supervised machine learning model was acceptable to patients and clinical team members. As both machine learning and direct patient outreach are novel methods for medical screening and diagnosis, considerable effort was dedicated to the design of outreach materials and manual chart review to increase program credibility among the community.

From this quality improvement work, 3 key lessons are applicable to future direct-to-patient FH outreach efforts: (1) Making the scheduling process as easy and efficient as possible for patients and clinical teams; (2) Establishing relationships with local primary care and maintaining communication with shared patients' PCPs (e.g., through referral notes); and (3) Prioritizing an informed, caring, and respectful tone in patient communications.

Patients highly valued outreach from physicians on our project team, but this outreach approach is unlikely to be sustained in practice due to competing demands for physician time. Drawing on the principles of design thinking, our effort prioritized the desirability and acceptability of the outreach program in support of service users' needs while paying less attention to its viability and the feasibility of maintaining it over time [25]. To address these issues, future work should consider team-based models to sustainably fill this outreach role. For example, a primary care-based health coach, nurse navigator, or community health worker conducting FH outreach would benefit from existing patient relationships while also facilitating sustainability by lowering the costs associated with high-touch outreach.

While modifications to specific design elements of our FH outreach program may further promote its effectiveness and sustainability, organizations providing health services in the US are incentivized to focus on treating rather than preventing illness. In the cardiology context, where interventions to treat acute disease are resource-intensive, a fee-for-service payment model incentivizes health systems to prioritize the use of these expensive interventions to treat disease rather than less expensive interventions to prevent disease and disease progression. The US policy environment therefore directs health system resources downstream to the acute setting. Financial models that instead prioritize population health through preventive care would improve the success and sustainability of programs like ours.

### 4.3 Conclusion

Partnership with the target population led to direct-to-patient FH outreach that was acceptable to patient recipients and to clinical team members. Repeat phone calls by lipid specialists maximized uptake of FH clinical evaluation, but this approach is unlikely to be sustained in routine practice.

## CRediT authorship contribution statement

**Rachel C. Forcino:** Methodology, Investigation, Formal analysis, Writing – review & editing, Writing – original draft. **Terry Sturke:** Investigation, Formal analysis, Data curation, Writing – review & editing. **Mary P. McGowan:** Investigation, Conceptualization, Writing – review & editing. **Amanda N. Perry:** Investigation, Formal analysis, Data curation, Writing – review & editing. **Shoshana H. Bardach:** Validation, Methodology, Writing – review & editing. **Vikrant S. Vaze:** Validation, Investigation, Writing – review & editing. **Kerrilynn C. Hennessey:** Investigation, Funding acquisition, Conceptualization, Writing – review & editing.

## Funding

This work was supported by the Susan and Richard Levy Health Care Delivery Incubator, a joint venture between the Dartmouth Institute for Health Policy & Clinical Practice and Dartmouth Health.

## Declaration of competing interest

The authors declare that they have no known competing financial interests or personal relationships that could have appeared to influence the work reported in this paper.
